# Barriers and enablers for oral health promotion programs amongst primary healthcare stakeholders in Qatar – a qualitative investigation

**DOI:** 10.1186/s12903-023-03633-4

**Published:** 2023-11-25

**Authors:** Asmaa Othman Alkhtib, Kamran Ali, Anand K. Sajnani, Lamyia Anweigi

**Affiliations:** 1 College of Dental Medicine, QU Health, Qatar University, Doha, 2713 Qatar; 2 Primary Health Care Corporation, Doha, Qatar; 3 Faculty of Medicine, Caucasus International University, Tbilisi, Georgia

**Keywords:** Oral health promotion, Inter-professional care, Qualitative study, Barriers to oral health promotion, Primary healthcare professionals

## Abstract

**Background:**

Oral health of preschool children remains a concern globally. Primary healthcare providers are in a vital position to support preventive oral care programmes. This study explored current practices, perception and barriers of primary health care professionals towards oral health promotion program of children in Qatar.

**Methods:**

The qualitative research used focus group discussions and interviewed a total of 108 participants that were audio recorded and transcribed verbatim. Four major themes emerged and were analysed to explore contextual patterns within the data.

**Results:**

Participants acknowledged the high prevalence of caries in children and identified the causes in the local context which included parental practices, poor dietary habits, impact of culture lack of oral health knowledge, limitations in the healthcare system, and negative role of the media. However, complex barriers were exposed, including lack of time and ownership, system coordination between organizations, and lack of policy.

**Conclusion:**

Health professionals and bureaucrats involved in decision-making held a positive attitude towards oral health prevention programs and were enthusiastic to initiate and support these programs.

## Introduction

Oral health is a core component of general health and can be described as a state where an individual is free of chronic orofacial pain, mucosal disease or cancer, craniofacial defects such as oral clefts, gum diseases, dental decay, tooth loss or any other disorders affecting oro-dental tissues [1, 2]. Dental caries is a major public health issue and can cause distress to young children due to a broad range of functional impairments including difficulty eating, sleeping, speaking, maintaining cognitive focus and controlling behaviour [3–5].

Despite the association between oral disease with systemic disease, the oral health of children remains a concern worldwide [4]. Whilst parents hold primary responsibility for maintenance of their child’s oral health, a number of stakeholders who are of relevance to preschool children’s oral health include primary health care professionals – dentists, paediatricians, health care directors, bureaucrats and administrators. Primary health care had been introduced as a gateway to address the equitable, population-centered service delivery and needs of a complex heath care system [6]. These primary care providers are well positioned to support preventive care and reduce the impact of a wide variety of oral conditions especially dental caries [3, 7, 8]. Therefore, it is imperative to understand their views as they are all in a position to have an impact on the quantity and quality of oral health care provided to preschool children.

Qatar has shown substantial progress in the quality of health care in the past 30 years and has the best Health care Access and Quality Index in the GCC (Gulf Cooperation Council) region [9](GBD 2015). In spite of these health care developments, oral health care remains a critical public concern for authorities in Qatar [10]. Several studies conducted in the GCC countries, including Qatar, have documented a high prevalence of dental caries in young children [11]. These studies have underscored the need for sustained community- based preventive programmes and professional care that should begin during pregnancy and early childhood [11, 12]. Routine Well Baby Clinics (WBCs) are established at Primary Health Care Corporation (PHCC) centres throughout Qatar and they serve as vital tools to promote children’s general health. All residents of Qatar have access to these primary health centres at exceptionally subsidised cost and their children can avail services of WBCs at these centres [12].

“Beautiful smile” project, an initiative of PHCC has been implemented across all primary health centres in the country to address the oral health needs of children. Dentists are key providers of this programme supported by other health care professionals. However, to achieve realistic progress on preventive programs to control dental caries in preschool children in Qatar and for the complete execution of the “Beautiful smile” project, it is imperative to assess the perceptions of health professionals employed at PHCC. Furthermore, it is necessary to comprehend the views of health bureaucrats and administrators who have the authority at various levels to facilitate or hinder health promotion activities particularly oral health related promotion activities. Currently, data available on acceptance of oral health promotion program amongst health care professionals is scarce [13–15], particularly in Qatar [12]. Therefore, the aim of this study was to explore current practices, perception and barriers of healthcare professionals in a primary care setting towards oral health promotion program of children living in Qatar.

The following research questions guided this study:

- What are the perceptions and experiences of health care professionals, administrators and bureaucrats regarding oral health care of children?
- How knowledgeable are the health care professionals, administrators and bureaucrats about oral health conditions, particularly dental caries?
- What are the perceived facilitators and barriers for health care professionals, administrators and bureaucrats to provide preventive oral health care and support oral health care promotion programs (“beautiful smile project”) for children in Qatar?

## Materials and methods

The study employed a qualitative research design using interviews and focus group discussions, including medical professionals, nurses, dentists and dental assistants at primary healthcare centres in Qatar. Purposive sampling was used to recruit the participants. A structured interview process was adopted to ensure that the information obtained from the participants can be assessed objectively. An interview guide was developed prior to the execution of the interview process. The guide was based on the objectives of the study and the published oral health status of children in Qatar [11, 12]. The guide provided in-depth information and identified personal experiences and context during interviews whilst it contributed to a plethora of information and opinions during the focus group discussion. Furthermore, both open and closed ended questions were included in the discussions so that the participants had the opportunity to contribute to the research topic questions. This methodology ensured that a true reflection of the social reality of the participants were recorded thus adding credibility to the study.

Interviews with health centre directors were conducted in their respective health centres while interviews with health bureaucrats were conducted at the headquarters of PHCC. All interviews went smoothly with great enthusiasm from interviewees. Focus group discussions involved medical practitioners, dentists, nurses and dental assistants. At the beginning of each focus group a brief account was given about the status of tooth decay in kindergarten children in Qatar, followed by an overview of the Beautiful Smile Project.

The interviews and focus group discussions were audio recorded and transcribed verbatim. The recordings and transcripts were stored on a password protected computer and participants were de-identified throughout transcription to ensure confidentiality and anonymity of the participants. The transcripts were individually read and re-read to gain familiarity. Subsequently, a comprehensive coding framework was developed.

A thematic analysis involving an inductive approach was used to identify and analyse contextual patterns and themes within the data. Data were coded to conceptual headings determined through open coding in the preliminary data analysis phase. Recurring terms and ideas were sorted into themes based on participant’s experiences. Debriefings were organised amongst the researchers of this investigations to discuss completeness of data. Any new areas to be explored were discussed until data saturation confirmed the analysis. This detailed approach ensured dependability of the study and consequently enhanced the rigor of the study.

## Results

There were 108 participants in this investigation, and the response rate of the health professionals was 100%. Demographics of key informants (KI) who participated in the interviews are provided in Table 1.

**Table 1:** Demographics of key informants (participants) recruited for interview

No.	Code	Position	Gender	Experience years
1.	KI-1	HB-1	Male	32
2.	KI-2	HB-2	Male	19
3.	KI-3	HB-3	Male	38
4.	KI-4	HB-4	Female	17
5.	KI-5	HCD-1	Female	19
6.	KI-6	HCD-2	Female	17
7.	KI-7	HCD-3	Male	23
8.	KI-8	HCD-4	Male	27
9.	KI-9	HCD-5	Male	13

KI = Key Informant, HB = Health Bureaucrat, HCD = Health Centre Director

Information about participants in the focus groups including their position, length of experience, gender and the number of participants in each focus group are detailed in Table 2.

**Table 2:** Demographics of health professionals (participants) recruited for focus group discussion

FG	MP	Nurses	Dentist	DA	Experience (years)	Gender	Total
FG-1	2	7	0	0	5–29	2 M, 7 F	9
FG-2	1	9	3	3	3–24	3 M, 13 F	16
FG-3	2	0	4	2	8–24	2 M, 6 F	8
FG-4	1	3	2	2	8–35	4 M, 4 F	8
FG-5	4	5	2	2	5–30	5 M, 8 F	13
FG-6	0	0	14	0	2–20	3 M, 11 F	14
FG-7	3	2	0	0	5–22	2 M, 3 F	5
FG-8	3	3	1	1	6–28	2 M, 6 F	8
FG-9	2	6	1	2	8–24	3 M, 8 F	11
FG-10	2	2	2	1	13–25	2 M, 5 F	7
Total	20	37	29	13	2–35	28 M, 71 F	99

FG = Focus group, MP = Medical Professionals, DA = Dental assistants

Despite the wide variation in the dynamics of the interviews and focus group discussions, several common themes were identified from the data. The thematic analysis of the interviews and focus group discussions uncovered interesting aspects about the perception of oral health problems and approaches to tackle them both administratively and practically. Four major themes from this qualitative work were identified:

- existence of dental caries issue in preschool children in Qatar,
- possible aetiology of dental caries in the Qatari context,
- enablers for the oral health promotion program (beautiful smile project) that may assist in managing the problem, and.
- possible barriers to the beautiful smile project.

Broadly, the barriers and enablers were categorized into three aspects: structural/ systemic, interpersonal and those based on social norms. These themes have been categorized in Table 3.

**Table 3:** Summary of structural, interpersonal and social enablers and barriers to the beautiful smile project

	Enablers	Barriers
Structural/ Systemic	Shared goals and vision and acceptance of the project Developing basic guidelines Expansion of health centres and planned new health centres	Lack of time and communication Limitation of health system facilities and current way of service provision Lack of training programs in oral health promotion
Inter-personal	Willingness to support oral health promotion programs Recognition of the importance of the project	Lack of skills in dealing with very young children Heath professionals blame parents and vice versa for oral health issues
Social norms	Parental and community education	Parental attitudes, beliefs, lack of appreciation and compliance Poor oral health knowledge

### Theme 1

#### Prevalence of dental caries in preschool children in Qatar

Participants acknowledged the dental caries in preschool children in Qatar was observed on a remarkable scale. All the health care practitioners conceded that most of their child patients had tooth decay and that majority of the children are afraid of doctors.

*“We have noticed a rise in tooth decay in preschool and school aged children. Unfortunately, our state has no initiative for the preschool category [children]… if a child has caries in one tooth in preschool [age], this may extend to many teeth by the time he reaches school” (KI-2)*.

*“I don’t see many children usually, but most of the one I see do have dental caries… it is very common that a child is not taken to a dentist unless he has unbearable pain or infection” (KI-5)*.

*“… sometimes they [children] complain about pain in their ears which later turns out to be [referred pain] because of a dental problem” (KI-6)*.

*“ Poor oral hygiene and dental caries in particular are very common problems among preschool children and extensively found during the preschool checkups [well baby check up]… Many times, we examine some children for illness like fever and discover that there is some dental abscess.” (KI-7).*

### Theme 2

#### Possible aetiology for dental decay in preschool children in the Qatari context

Collectively, participants identified several causes for dental decay in the Qatari context - parental practices and attitudes towards oral health including poor dietary habits, the impact of culture, lack of oral health knowledge and oral health education, limitations in the health care system, and the negative role of the media in promoting extensive advertisement of unhealthy foods for children.

### Parental practices and attitudes towards oral health

Interviewees indicated that parental practices and attitudes towards oral health played a significant role in causing dental decay in preschool children. The main practice identified was inappropriate feeding habits.

*“In this part of the world, the parents have enough wealth to provide their children whatever they demand. I think that wealth along with lack of health education add to the dental problems” (FG-6)*.

There was also a general consensus in the focus groups that families and particularly mothers were responsible for caries in their children because they were the primary caregiver for the child. They did not provide appropriate care for the oral health of young children due to the general perception that primary teeth are temporary, not important and will be eventually replaced. The lack of positive attitude towards the dentist was another factor in causing dental decay.

*“…the oral health of the child is the responsibility of their parents. If parents are not caring, the child comes to the dentist at very critical [dental] stage, which makes it difficult for us to treat them…” (KI-1)*.

Some participants claimed that when families do visit the dentist they are anxious during the appointment and often leave the clinic once the child started crying. Many health professionals did not take any professional responsibility for the problem. Some even claimed that children have healthy diets but lazy parents who failed to take their children for regular dental check-ups.

### Lack of oral health knowledge and awareness

Lack of oral health knowledge and awareness in the community was another aspect identified by interviewees as a contributory factor in causing dental decay in preschool children. The participants explained that due to lack of health education, particularly oral health education, families do not teach their children about healthy food choices. They perceived that the parental neglect is due to insufficient health education.

*“It may be due to lack of education about oral heath because many parents don’t have enough knowledge about importance of brushing… They think that the milk teeth will eventually be replaced by permanent teeth so the milk teeth are not very important” (KI-9)*.

*“I think one of the causes is the lack of awareness among the parents about the healthy diet for the child.” (FG-9)*.

### Cultural practices

Participants indicated that cultural practices were influential in causing dental decay in preschool children. The practice of leaving the responsibility of feeding the children to the maid and providing too many sweets to children as a way of indulgence is a common culture in Qatar.

*“…feeding (children) is usually left to the maids…they use sweets and chips to keep children calm…mothers don’t monitor the child’s teeth or follow-up with the dentist…” (KI-1)*.

*“The most common reason is the bad feeding habits and making sweets available to the children at all times. Another reason is that the children fear the treatment procedures and the pain caused by them, and eventually stops seeing their dentist” (FG-7)*.

*“The most prevalent problem about oral health of children is dental caries because of the sweets, fast food, carbonated drinks etc which the children eat in big portions. If a mother feels that the child is thin, she will start feeding him sweets to gain weight” (KI-6)*.

Furthermore, parents are not inclined to visit the dentist for their children’s oral health issues.

*“Parents are always in a hurry and lack patience; they may end the meeting with the dentist if the child starts crying”. (FG-5)*

### Limitations in the health system

Participants felt that the current health care system in Qatar has limitations that may contribute to the problem of dental decay in preschool children. The current practices of health professionals (excluding dentists) did not necessarily include oral health check-ups or advice.

*“During my period of 2 years working here as a dentist, I have never received any referral from the Well Baby Clinic” (FG-4)*.

*“After the injection [immunisation], we might provide some information but not about oral health necessarily.” (FG-1)*.

*“No we don’t do that [examine a child’s teeth in the WBC] except if they are complaining about something” (FG-8)*.

Besides, there is a paucity of dedicated oral health services for preschool children who are at the greatest risk of developing dental caries

*“Unfortunately, our state has no [oral health] initiative for preschool category. We lack programs that are directed towards the families and kids to direct them about their dental hygiene and proper dental care… we are lacking an authorized body to monitor this issue [oral health of preschool children]” (KI-2)*.

### The negative role of the media

Participants felt that uncontrolled media promotion for unhealthy food plays a substantial role in the dental decay problem. Several health professionals thought that the media has a major responsibility for the problem and them advertisement responsibly. They claimed that media increasingly promotes unhealthy food such as candies, which plays a significant role in increasing the problem.

*“The media and outdated education have created an impression in parent’s minds that dentists should be visited only if a dental problem happened” (HB-1)*.

*“I think the media can at least stop promoting unhealthy food.” (FG-4)*.

### Theme 3

#### Enablers for the beautiful smile project that may assist in managing the problem

The interviewees identified several ways to manage the problem of dental decay in preschool children.

### Interpersonal enablers

There was a positive attitude towards the Beautiful Smile Project. All participants supported the proposed project to manage the dental decay problem in Qatar.

*“…all primary and semi-primary, governmental and private sector should work together to make this project successful” (KI-2)*.

*“If we have an approval from the higher authority with a budget I think things will be easier” (FG-8)*.

*“We [in this health centre] provide pre-natal and post natal advice about teething, eruption, oral hygiene etc [but] this is not a limited problem within the dental community, but it is a major problem and everyone should contribute to solve it, dentists, general doctors, parents and media… it must be a mass campaign” (KI-7)*.

Some participants felt the need for change and improvement of the current situation in relation to oral health careof preschool children and acknowledged the importance of good oral health for children. They stressed the importance of looking after the children’s teeth and providing them with good “feeding” habits as they represent the future parents of Qatar.

*“We need to have separate facilities for children with people trained to handle these children which we actually cannot find here” (KI-1)*.

*“First we need to have space in the PHCC. This matter should be taken into consideration when they are planning the layout for the new PHCC. We also need to have the personnel who will be taking care of all these activities. We need to have time and the budget for the toys, equipment etc.”(KI-4)*.

Furthermore, participants believed that the way health care practitioners deal with children during an appointment was critical for cooperation from children for further evaluation and treatment.

“*The first visit to the dental clinic should not be confined to treatment; instead it should be spent in obtaining the child’s trust by “nice” gentle management using small gifts as a reward for cooperation*” (FG-7).

### Structural enablers

There were several categories identified as structural enablers: shared vision and goals, increasing capacity of the staff and optimising the role of the media.

- Shared goal and vision.

There was an anticipation of success for the beautiful smile project because it fits the new vision of primary health care for Qatar. Interviewees highlighted the importance of collaboration within relevant departments at PHCC and between PHCC and all other agencies within the health sector in Qatar.

*“I would like to stress the need for collaboration…the primary health care unit cannot function alone, Hamad Medical Corporation (HMC) a government health institution, Ministry of Public Health and Sidra [health institution] etc need to be a part of this project. All the primary, semi primary, government, private sectors should work together to make this project successful.” (KI-2)*.

*“The Supreme Council of Education has to arrange with the professionals to conduct health education sessions for children and the teachers also because it happened that sometimes when we provide important information to the children, the teachers don’t know it also…” (FG-4)*.

Furthermore, they stressed the importance of teamwork as well as cooperation between dental clinics and the WBCs within each health centre in order for the initiative to be successful.

*“There should be effective communication between different clinics [within the health centre]” (KI-3)*.

- Increase capacity of staff to manage preschool children and create supportive environments.

Most participants agreed that it was imperative to improve the capacity of the current staff working in PHCC to manage preschool children. Health professionals need to be recruited and oral health promotion has to be imparted to all staff dealing with children to improve their efficiency. Participants stressed the importance of ongoing professional development workshops in all health centres.

*“First…we should…recruit suitable dentists to see preschool children because not everyone has patience and capability…Dentists should attend workshops…to enhance their skills…we can dedicate some times of our existing staff like nurses, paediatricians and dentists…and helping them in promoting these ideas…” (KI-1)*.

*“We need to share our knowledge. The general doctors need to learn about the dental problems in the same way the dentists need to learn about other diseases. So I think we do need to have joint sessions periodically.” (FG-3)*.

- Optimising the media.

Interviewees highlighted the importance of the media’s role in promoting oral health. They suggested that media should play a role in health promotion by streaming educational programs about oral health for families.

*“I believe that we strongly need the support of media for creating awareness. Through media, we can deliver the message easily and have a greater impact…” (KI-5)*.

*“Media needs to play a more effective role to guide the parents about good dietary habits for their children” (FG-10)*.

*“We can also disseminate the message to the community through campaigns in shopping malls, centres and through media like newspapers, radio, TV and modern social media like face book, twitter which are widely used by the new generation” (KI-4)*.

### Social norms enablers

- Parental and community education.

Participants stressed the significance of community education in improving the oral health of pre-schoolers. They suggested that specifically targeted initiatives will aid in improvement of the oral health status of pre-schoolers in Qatar and improve oral health knowledge of parents.

*“We first should provide necessary education to patients to bring about awareness” (KI-3)*.

*“… If they [the community] knew that the milk teeth are as important as the permanent teeth and in future may affect the other functions like speech etc then they would definitely call for this service.” (KI-2)*.

*“The mother should have direct and continuous supervision over her child so that she knows everything about him.” (FG-2)*.

Some participants highlighted the importance of the introduction of educational and tooth brushing programs in schools and sourcing fund for such programs.

*“We would always like the message to reach the whole community and schools are a part of it. Many channels can be reached indirectly like families can be reached through students in schools because two-thirds of the population is in schools… We could give orientation in schools as we can reach the family through the child…” (KI-4)*.

### Theme 4

#### Barriers to the project

Collectively, the participants identified several potential barriers that may challenge the implementation of The Beautiful Smile Project. These barriers fall into three categories: interpersonal, structural and social norms.

### Interpersonal barriers

Participants anticipated that parents might not appreciate the importance of the Beautiful Smile Project.

*“Parents may not appreciate the importance of such programs… We are already facing difficulties in getting the parents to bring their infants for getting their vaccinations” (KI-3)*.

In response to a query about claims that mothers take their preschool children to visit the dentist but are turned away for different reasons, the participants responded with disagreement.

*“I don’t think that the dentist refuses to treat a child. Of course, they will try to avoid invasive procedures as the child is scared and also try to minimize interference as much as possible. Advice and medications will definitely be provided whenever required” (KI-8)*.

*“I don’t think so because no one will be denied treatment in the PHCC except if there are genuine reasons like absence of doctor, unavailability of appointment slot [number in the queue to see the dentist] etc because as you know we follow first come-first serve basis for walk-in appointments” (KI-4)*.

Most health care practitioners (except dentists) conceded that they provided little to no information on oral health for their child patients.

*“We do give advice but it may not be well structured. For example, we may tell a family that their child has a cavity and they have to see a dentist, but we don’t provide a specific advice regarding prevention or management” (KI-2)*.

*“No actually we don’t. We do provide them advice for other problems like under growth, underweight etc but we don’t concentrate on oral health.” (KI-6)*.

*“In my experience, I didn’t see many doctors caring about the oral hygiene specifically and I feel there is shortage in this field because only paediatricians study oral hygiene but other medical doctors don’t… A person’s teeth can be a mirror for underlying health problems because many diseases can be diagnosed from oral signs. The general doctors are not aware of such diagnoses. I feel that this is the area where most of us lack knowledge” (KI-7)*.

*“In general practice this [dental check-up] is not happening maybe because of lack of time allocated to examine each patient or sometimes the doctor forgets it…” (KI-9)*.

Some interviewees felt the Beautiful Smile Project might face challenges upon implementation because it is a drastic change from the norm.

*“…dentists may fail in managing preschool children and refer them to HMC creating referral overload…well baby clinic staff may fail to examine cases [children] due to the new concept…parents may think a preschool child is too young to see a dentist…” (KI-3)*.

*“I feel that there will be a difficulty in implementing it but as you know, everything is difficult when it starts to be implemented but people will cope themselves in time” (KI-5)*.

### Structural or systemic barriers

Structural barriers identified included shortage of staff, lack of space, lack of appropriate training and continuing professional development of the staff. There were no special clinics for children and the existing clinics were not child-friendly. Furthermore, there was a lack of oral health promotion programs and there was no authorizing body (for instance a dental association) to plan and monitor such programs, and to gain cooperation from higher authorities.

- Limitations of the health system facilities and current way of service provision.

Almost all participants admitted the limitations in the current health system in providing focused dental care for preschool children. Health facilities lack well defined systems for child oral health care. There is a lack of public health programs (especially oral health) provided by the Ministry of Public Health.

*“Population explosions in recent years created a lot of pressure on the primary health sector….primary health centres are already overloaded…” (KI-2)*.

*“… The clinics in the health centres are designed to just cover the needs of the society. If we try to designate other clinics on behalf of the current clinics, it may lead to inconvenience in the society” (KI-3)*.

*“The dental clinics are very busy. The numbers [of patients] allocated to these clinics are distributed within 15–20 min. If we allocate one of the available clinics to preschool children only, it will result in shortage” (KI-5)*.

Participants in focus groups acknowledged that the time allocated for each patient is not enough for dental treatment and family education. Limited time available for each patient was a big hurdle in providing optimal oral hygiene instructions and other oral health promotion advice by dentists.

*“We know that the dentists are busy and even if this [referral from the well baby clinic] happens the dentist may talk to the parents for less than a minute and see the other patients who he believes need more attention than this healthy child” (FG-1)*.

*“Yes we need about 30 minutes to control the child and treat if they are cooperative. It may take more time if they are not cooperating.” (FG-10)*.

- Staff number and quality of training.

All key informants stressed the issue of staff shortage and the challenges involved in recruiting staff. They also highlighted the lack of appropriate continuing professional development to improve skills of the current staff to implement such initiatives.

*“There are barriers facing dentists in seeing very young age [children], every person have different nature… we need trained people to handle these children, which we actually cannot find here” (KI-1)*.

*“We are suffering from manpower limitation to establish such dedicated clinics… I think the training that has been given to our dentists does not give them the total experience to treat preschool children.”(HB-2)*.

- Social norms barriers.

Participants identified several social norms barriers including cultural barriers, and lack of health knowledge which was reflected in poor compliance with the health advice and consequently late presentation of disease complications.

*“Lack of knowledge in the families about the dental health and their feeling that there is no harm in decay or cavity of milk teeth has made them reluctant in pushing the authorities to have Community Paediatric Dentistry” (KI-2)*.

*“The parents’ approach plays a very improtant role. They must not feel that a child is too young to see a dentist at that age. Also many mothers think that these temporary teeth (milk teeth) will be sooner or later replaced so they are not aware of their importance” (KI-3)*.

*“I notice that most of the parents do not come to the dentist except after the tooth is totally destroyed. They don’t check their children’s teeth regularly for any dental caries or other problems.” (KI-9)*.

## Discussion

Oral health of children has been a pressing issue in Qatar and tailored approaches to prevention are warranted. Qualitative inquiry typically focuses in depth on relatively small purposefully selected samples. This investigation used a qualitative approach to get a deeper insight into the perception, understanding and experiences shared by the participants who were key stakeholders in PHCC in Qatar [6, 16].

The results of this study revealed intriguing insights. There was a general agreement among the participants about the significance of the early childhood caries problem. However, there was a wide variation in the understanding of the aetiology and the risk factors for early childhood caries. Many health professionals, dentists and dental assistants in particular had described the problem from a pathological perspective while the other health care professionals approached the problem broadly from a socio-biological perspective. The pathological model focuses on the bacterial factors and oral hygiene level in contrast with the socio-biological model, which provides a holistic view of the health problem in all its complexity. The health bureaucrats often use an organisational and systemic lens to view of the problem. It is well documented that early childhood caries is a complex health problem that is influenced by social, psychological, economical, cultural and environmental factors in addition to the biological and pathological factors [17–21]. Irrespective of the view held, all health care practitioners found it challenging to address the oral health hygiene promotion.

Challenges faced for oral health promotion integration included lack of time and appropriate facilities, insufficient staff, poor compliance from parents and lack of administrative support which was concordant with other studies [22, 23]. Studies from Sweden [24], Spain [25], and USA [26], have indicated that health care professionals at the primary level are inclined to provide preventive treatment but they face severe constraint in the form of limited time and resources due to an overload of curative care. Interviewees unanimously accepted constrained time for oral health care instructions. Lack of time has been recognised as the most challenging barrier to provide oral health services/ information by primary health care providers [27, 28].

For some, such as medical practitioners and nurses, it was a minor issue as they were occupied with their busy jobs. In the Qatar primary health care setting, it is not common for non-dental health professionals to provide oral health promotion services. Alternatively, for the dental team, dental caries in pre-school was a major issue, but the overwhelming amount of workload to treat the dental caries left them with very little time to address the preventive aspects or promote oral hygiene. Nevertheless, all participants unanimously agreed that recruitment of skilled staff specifically; dentists and dental assistants will provide a viable and efficient means to address this issue. Administrators and bureaucrats need to channelize the available resources effectively towards identifying and enrolling proficient health care practitioners in this field.

This qualitative investigation strongly identified a “victim-blaming” attitude from health professionals. It has been well documented that the “victim-blaming” concept has been prevalent in the health service delivery in western medicine and still exists [29, 30]. In the current study, almost all interviewees blamed the parents, particularly mothers, for the early childhood caries problem. The criticism ranged from indulging children and leaving children in the care of nannies to lack of appropriate oral health knowledge. Nevertheless, a few health professionals acknowledged the shortcomings of the health system and submitted that the health system and the media played big roles in the early childhood caries problem in Qatar. Blaming the parents and the media reflects disowning the problem rather than claiming a share in responsibility as health professionals who did not meet their health promotion duties. Alternatively, some participants felt that all who deal with the child share responsibility regarding their health, including the family, the school and the health professionals.

It is difficult to ascertain the impact of mothers’/ parents’ inappropriate oral health practices and attitude on children’s oral health. There is some evidence that having good health knowledge may not necessarily dictate good health choices and practices [31]. On the contrary, some studies reported that poor oral health literacy was associated with poor oral health choices such as nocturnal bottle feeding and frequent provision of cariogenic snacks [32]. Whilst it is normally perceived that an individual cannot give what s/he does not have, there are many people who teach good practices despite the fact that they still indulge in poor practices themselves [33–35] .

Nevertheless, oral health promotion and prevention is a multidisciplinary issue in both its implementation and consequence. Dental caries is preventable by simple activities like increasing awareness and education through health promotion activities, as risk factors include poor dietary habits, poor child feeding habits and poor oral hygiene practices. Oral health promotion is based upon the idea of educating people with relevant knowledge so that they may develop the motivation and the understanding in order to change their behavioural patterns regarding oral health care [36, 37].

Some participants of this investigation had never participated in any research before and for most of them, it was their initial experience with focus groups. This may have had a subconscious impact on their degree of interaction. Moreover, a small group of participants had crucial roles in the organisation (PHCC) which perhaps had an impact on their views. They may have concealed some information and failed to recognise or admit shortcomings to avoid being in a vulnerable position. Results from qualitative research cannot be generalised to an entire population. However, given the fact that this study contained a very large number of participants from the only primary health care organization in the country and achieved data saturation, the results of this study cannot be underrated. Moreover, there are no other studies conducted on this subject in Qatar, the findings of this study are valuable and provide an insight into the barriers and facilitators for oral health promotion amongst primary health care professionals. Furthermore, the results of this study are consistent with studies conducted in other primary health care settings and thus potentially have implications for oral health care promotion worldwide [12, 38–41].

Barriers identified in this study need to be addressed effectively in order to integrate oral health preventive services into the primary health care setting. This investigation demonstrates that efforts toward interdisciplinary team-based collaboration can be complicated by professional boundaries drawn by professional values, beliefs, attitudes, customs and behaviours [42, 43]. A small group of participants were cautious in discussing their ideas and perception possibly due to cultural boundaries, job insecurity and lack of trust, or perhaps for the fact that participating in research was a new concept for them. Nevertheless, clinic staff unanimously agreed that a committed vision from top administrators, designated team leaders, were necessary for meaningful change to occur.

Oral health promotion programmes are often influenced by cultural perspectives while also addressing the social determinants of health [44–46]. Health provider’s recommendation that paediatric patients visit dentist, has been associated with increase in dental visits among 2 to 5 year-old children [40]. The Beautiful Smile Project can benefit manifold if healthcare practitioners can adhere to this recommendation. One potential method to implement this practice would be to have a dedicated time on a weekly basis where the dental department would update the entire team on programs and strategies to combat dental caries in children. Furthermore, there can be a provision in the electronic medical record system of patients where the healthcare practitioner has to record the oral health status of the patient failing which the patient data cannot be saved on the system. Furthermore, relevant authorities and bureaucrats need to rope in specialist preventive and paediatric dental personnel in the program to improve its success rate. Potentially effective interventions ought to be performed during the first two years of a child development [17]. However, traditionally dental attendance before 2 years of age is uncommon even though attendance with other health professionals is high [47]. The state media can positively impact to reserves this phenomenon by promoting the aforementioned changes in the structure of oral health services at PHCC under the Beautiful Smile project.

The widespread neglect of oral health of preschool children in Qatar has been well documented [11]. This scenario and the findings from the current study make it imperative to introduce concrete, effective and uninterrupted preventive programmes particularly at the Primary Health care level. Various opportunities to deliver such programmes exist in the primary health care system and the results of this study unequivocally indicate that health care providers are willing to support this cause. However, it is vital that administrators, bureaucrats and people in the administrative position propagate oral health promotion programs vigorously.

## Conclusions

Health professionals, administrators and bureaucrats involved in decision-making shared a positive attitude towards oral health prevention programs and were enthusiastic to initiate and support these programs. Complex barriers including lack of time and ownership, lack of system coordination between organizations and lack of policy were exposed and constructive avenues were highlighted to initiate positive changes into the system. This study provides immense support to the oral health promotion programs amongst primary health care professionals and potential for widespread application.

## Data Availability

The datasets generated and/or analyzed during the current study are not publicly available due to the research policy of the institution of the principal investigator but are available from the corresponding author on reasonable request.
